# Digital health in stroke: a narrative review

**DOI:** 10.1055/s-0044-1789201

**Published:** 2024-08-26

**Authors:** Gisele Sampaio Silva, João Brainer Clares de Andrade

**Affiliations:** 1 Universidade Federal de São Paulo, Departamento de Neurologia, São Paulo SP, Brazil.; 2 Hospital Israelita Albert Einstein, Organização de Pesquisa Acadêmica, São Paulo SP, Brazil.; 3 Instituto Tecnológico de Aeronáutica, Laboratório de Bioengenharia, São José dos Campos SP, Brazil.; 4 Universidade Federal de São Paulo, Departamento de Informática em Saúde, São Paulo SP, Brazil.

**Keywords:** Digital Health, Stroke, Saúde Digital, Acidente Vascular Cerebral

## Abstract

Digital health is significantly transforming stroke care, particularly in remote and economically diverse regions, by harnessing mobile and wireless technologies, big data, and artificial intelligence (AI). Despite the promising advancements, a notable gap exists in the formal clinical validation of many digital health applications, raising concerns about their efficacy and safety in real-world clinical settings. Our review systematically explores the landscape of digital health in stroke care, assessing the development, validation, and implementation of various digital tools. We adopted a comprehensive search strategy, scrutinizing peer-reviewed articles published between January 2015 and January 2024, to gather evidence on the effectiveness of digital health interventions. A rigorous quality assessment was conducted to ensure the reliability of the included studies, with findings synthesized to underscore key technological innovations and their clinical outcomes. Ethical considerations were meticulously observed to maintain data confidentiality and integrity. Our findings highlight the transformative potential of mobile health technologies, AI, and telemedicine in improving diagnostic accuracy, treatment efficacy, and patient outcomes in stroke care. Our paper delves into the evolution and impact of digital health in cerebrovascular prevention, diagnosis, rehabilitation and stroke treatment, emphasizing the digital health's role in enhancing access to expert care, mitigating treatment delays and improving outcomes. However, the review also underscores the critical need for rigorous clinical validation and ethical considerations in the development and deployment of digital health technologies to ensure their safe and effective integration into stroke care practices.

## Resumo

A saúde digital está transformando significativamente o cuidado com o acidente vascular cerebral (AVC), especialmente em regiões remotas e economicamente diversas, ao aproveitar tecnologias móveis e sem fio, *big data* e inteligência artificial (IA). Apesar dos avanços promissores, existe uma lacuna notável na validação clínica formal de muitas aplicações de saúde digital, levantando preocupações sobre sua eficácia e segurança em ambientes clínicos do mundo real. Nossa revisão explora sistematicamente a paisagem da saúde digital no cuidado do AVC, avaliando o desenvolvimento, validação e implementação de várias ferramentas digitais. Adotamos uma estratégia de busca abrangente, examinando artigos revisados por pares publicados entre janeiro de 2015 e janeiro de 2024, para reunir evidências sobre a eficácia das intervenções de saúde digital. Uma avaliação de qualidade rigorosa foi conduzida para garantir a confiabilidade dos estudos incluídos, com os achados sintetizados para destacar as principais inovações tecnológicas e seus resultados clínicos. Considerações éticas foram meticulosamente observadas para manter a confidencialidade e integridade dos dados. Nossas descobertas destacam o potencial transformador das tecnologias de saúde móvel, IA e telemedicina em melhorar a precisão diagnóstica, a eficácia do tratamento e os resultados dos pacientes no cuidado do AVC. Nosso artigo aprofunda-se na evolução e impacto da saúde digital na prevenção, diagnóstico, reabilitação e tratamento do AVC, enfatizando seu papel em melhorar o acesso ao cuidado especializado, mitigar atrasos no tratamento e melhorar os desfechos. No entanto, a revisão também sublinha a necessidade crítica de validação clínica rigorosa e considerações éticas no desenvolvimento e implantação de tecnologias de saúde digital para garantir sua integração segura e eficaz nas práticas de cuidado do AVC.

## INTRODUCTION

Digital health has emerged as a transformative force in managing stroke, a leading cause of morbidity and mortality worldwide—especially when we consider remote and low- and middle-income areas. 1 By leveraging advances in mobile and wireless technologies, big data analytics, and artificial intelligence (AI), digital health can potentially improve stroke prevention, diagnosis, treatment, and rehabilitation. 1 2 Over the past decade, several landmark trials have demonstrated the efficacy and safety of digital health interventions in stroke care. 1 2

Digital health has the potential to revolutionize stroke care and improve patient outcomes. One of the most promising digital health applications in stroke care is telemedicine, which enables remote consultation and diagnosis by stroke specialists. 3 Telestroke programs have been shown to reduce treatment delays, improve clinical outcomes, and increase access to expert stroke care, especially in underserved areas. 3

A major challenge in the adoption of these digital health solutions is the lack of formal clinical validation for many of these applications. Despite the rapid development and deployment of digital health tools in the stroke field, there is a significant gap in the rigorous assessment of their efficacy and safety. This shortfall raises concerns about the reliability and effectiveness of these technologies in real-world clinical settings, where the stakes are high. The absence of robust validation processes means that the impact of these digital tools on critical clinical outcomes such as survival rates, functional independence, and quality of life for stroke patients remains unclear.

This lack of clarity regarding the clinical outcomes associated with digital health applications in stroke care underscores the necessity for a more structured and evidence-based approach to their development and implementation. It is essential to bridge the gap between technological innovation and clinical validation to ensure that these digital solutions can truly benefit patients and healthcare providers. The role of digital literacy becomes paramount in this context, as it empowers both clinicians and patients to navigate, evaluate, and effectively integrate digital health technologies into routine stroke care practices.

Our main objective is to provide a comprehensive narrative review of the current digital health applications available in the field of vascular neurology. By systematically evaluating the existing digital tools, their development processes, clinical validations, and real-world applications, we aim to identify the gaps in current practices and discuss the integration of digital health technologies in stroke care.

## METHODS

In this narrative review, we meticulously explored the role of clinically validated digital health applications in stroke patients, focusing on the integration and impact of these technologies in clinical settings. Our methodology was structured to encompass a comprehensive search and analysis of both review articles and original research that discuss digital health solutions and stroke.

### Search strategy

To identify relevant literature, we employed a combination of keywords and medical subject headings (MeSH) terms tailored to capture the breadth of digital health applications pertinent to stroke management. Our search descriptors included *digital applications* , *automated algorithms* , *clinical decision support platforms* , *web-based platforms* , *artificial intelligence* , and *mobile applications* , paired consistently with the fixed term *stroke* . These terms were used to search databases for articles published in English from January 2015 to January 2024, ensuring the inclusion of the most recent and pertinent studies in the field.

### Study selection

The inclusion criteria were set to select peer-reviewed original research articles and comprehensive review papers that specifically addressed the development, validation, implementation, and impact of digital health technologies in the context of stroke. Exclusion criteria were applied to omit studies that did not focus on clinically validated tools, were not available in full text, or were outside the specified publication date range.

### Quality assessment

To ensure the credibility and relevance of the included studies, a rigorous quality assessment was conducted. This evaluation was based on predefined criteria that considered the study design, methodology, sample size, bias, and the strength of findings. Each study was independently reviewed by two members of the research team, with discrepancies resolved through discussion or consultation with a third reviewer.

### Data synthesis

Data extracted from the selected studies were synthesized to highlight key findings, technological innovations, clinical outcomes, and the overall impact of digital health applications on stroke management and patient care. A narrative synthesis approach was adopted to accommodate the diverse nature of the studies.

### Visualization tools

To effectively summarize and present the vast array of digital applications identified in our review, we developed a detailed figure and a mind map. These visual tools were designed to categorize the applications based on their functionality, target user (healthcare professionals versus patients), and the aspect of stroke care they address (prevention, acute management, rehabilitation).

### Ethical considerations

Throughout the review process, ethical considerations were meticulously observed. The study was conducted in accordance with the ethical standards of the Declaration of Helsinki and was exempt from institutional review board approval due to its nature as a secondary analysis of publicly available data. Care was taken to ensure the confidentiality and anonymity of the data extracted from the studies included.

In our endeavor to enhance the efficiency and accuracy of our work, we have adopted the ChatGPT 4.0 generative AI tool (OpenAI, San Francisco, CA, USA), leveraging its capabilities for tasks such as translating from Portuguese to English, reviewing grammar mistakes, making textual adaptations, and generating images for the creation of mind maps through the ChatMind tool (Xmind Ltd., Hong Kong, China). This decision was made with careful consideration of the ethical implications associated with the use of advanced AI technologies. We ensure that all data processed through ChatGPT 4.0 and ChatMind are handled with strict confidentiality and in compliance with applicable data protection laws. We do not use these AI tools to process sensitive or personal information without appropriate consent and safeguards.

## DIGITAL HEALTH APPLICATIONS

Mobile health technologies such as smartphone applications, wearables, and sensors offer exciting opportunities for remote monitoring of patient's vital signs, medication adherence, and rehabilitation progress, enabling personalized and proactive management of stroke patients. 4 These applications have been developed to enhance diagnosis, treatment, and management of stroke patients, leading to better patient outcomes. Studies have shown that the use of mobile applications in stroke care has led to improved clinical outcomes, reduced treatment time, and improved patient satisfaction. 4 Some of these apps use AI algorithms to analyze symptoms and medical records to identify patients who are at high risk of stroke—and even differentiate those who may benefit from specific reperfusion therapies. These applications help healthcare providers quickly assess the patient's condition and initiate treatment, improving the their chances of a successful outcome. 1 4 5

Machine learning algorithms can help analyze large datasets of medical images, patient records, and clinical trials, leading to more accurate diagnoses, personalized treatment plans, and more efficient drug discovery, incorporated to complex platforms and even medical devices (such as monitor vital signs, tomography machines and others). 4 5 Still, it is critical to ensure these technologies are validated, safe, and accessible to all stroke patients, regardless of their socioeconomic status or geographical location. 1

In the field of vascular neurology, digital health modalities have diversified, offering a broad spectrum of technologies aimed at enhancing stroke care and rehabilitation. Telestroke services have become a cornerstone, enabling remote consultations and assessments through video conferencing, which is pivotal in acute stroke management, in which time is of the essence. This modality allows for rapid decision-making, often facilitating timely interventions such as thrombolysis or thrombectomy. Furthermore, mobile health applications and wearable devices are increasingly utilized for patient monitoring and management, offering features such as medication reminders, lifestyle modification tips, and real-time monitoring of vital signs. These tools support continuous patient engagement and self-management, which are crucial for secondary stroke prevention and rehabilitation.

Beyond telemedicine and mobile health, advanced digital platforms incorporating AI are emerging within the vascular neurology landscape. Artificial intelligence-driven algorithms are being developed to enhance diagnostic accuracy by interpreting imaging studies, predicting stroke risk, and personalizing rehabilitation protocols based on patient-specific data. Virtual reality (VR) and augmented reality (AR) technologies are also being explored for their potential in stroke rehabilitation, providing immersive, adaptive environments for patients to engage in therapeutic exercises. These modalities aim to improve motor skills, cognitive function, and overall recovery outcomes by simulating real-life activities and feedback in a controlled setting. Collectively, these digital health modalities are reshaping the approach to stroke care, offering innovative solutions to traditional challenges in diagnosis, treatment, and rehabilitation in vascular neurology.

**Figure 1:**
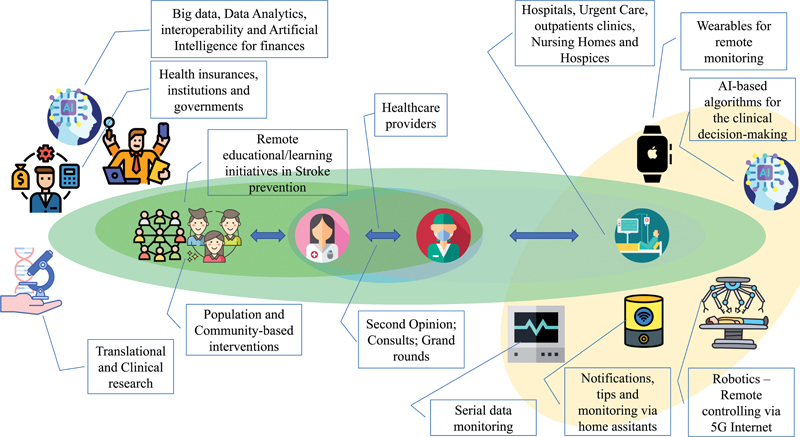
Abbreviation: AI, artificial intelligence. Modalities of technologies in digital health.

In Figure 1 , we summarize many of these modalities of technologies in digital health. This figure illustrates the diverse range of technological modalities utilized in the digital health landscape. It categorizes these technologies based on their application areas, including telehealth, mobile health apps, wearable devices, electronic health records, and AI in healthcare. Each category is represented with examples and icons that reflect their use in clinical settings, patient monitoring, data management, and decision support systems.

## CURRENT EXAMPLES OF DIGITAL HEALTH INTERVENTION AND IMPLEMENTATION IN STROKE

Digital health solutions are available to support both healthcare professionals and patients throughout all stages of stroke. These solutions have been designed to provide access to necessary treatments and therapies, such as intravenous thrombolysis and thrombectomy in acute settings and rehabilitation in poststroke care. 2 Additionally, various applications are available to monitor vital signs and encourage patients to adhere to their prescribed treatments.

In the field of secondary prevention, for example, a very interesting initiative for improving the adherence to oral anticoagulants has been published in the literature. 6 According to the authors, the app/intervention adopts reminders, educational material, and monitoring features to help patients manage their medication regimen and reduce the risk of stroke and other complications associated with atrial fibrillation. The app sends reminders to patients to take their medication, provides educational material about the importance of medication adherence, and allows patients to track their international normalized ratio (INR) levels, providing feedback and guidance to improve adherence. The study concludes that 72% of the patients in the low adherence group moved to either the medium or high adherence group. 6 The use of a smartphone app can significantly improve oral anticoagulation adherence in patients with atrial fibrillation and reduce the risk of stroke and other complications. The results suggest that mobile health technologies could play a valuable role in improving medication adherence and disease management in patients with chronic conditions.

Telemedicine enables patients to connect with healthcare professionals remotely, providing timely access to medical consultations and enabling doctors to assess stroke patients' conditions quickly. 1 7 8 The Stroke Telemedicine Outcomes Project (STOP) showed that telemedicine consultations could effectively reduce the time to treatment with thrombolytic therapy in patients with acute ischemic stroke. 9 The study involved 12 hospitals across California and demonstrated that telestroke consultations were associated with significantly higher rates of thrombolytic therapy administration within the recommended time frame compared with standard care. 9 This and other trials paved the way for the widespread adoption of telestroke programs, which have improved access to expert stroke care, reduced treatment delays, and improved clinical outcomes. 1 7 8 9

Telemedicine also allowed the incorporation of mobile stroke units (MSUs) in our clinical practice. Even restricted to a small number of areas, these vehicles are equipped with diagnostic and treatment equipment for stroke patients. These units have become increasingly important in improving clinical outcomes by providing faster and more efficient stroke care. 10 The MSUs can be deployed to remote or underserved areas, where there may be limited access to stroke specialists or stroke centers, thereby reducing the time to treatment and improving patient outcomes. 10 The use of MSUs enables patients to receive timely diagnosis and treatment, which may be crucial in the stroke care. With the availability of computed tomography (CT) scanners, laboratory testing equipment and telemedicine with an experienced neurologist onboard, MSUs can provide immediate diagnostic tests, and stroke treatment can be initiated in the prehospital setting, even before patients arrive at the hospital. This rapid response time reduces the time to treatment and leads to better clinical outcomes, including reduced disability and mortality rates. 11 In addition, MSUs can facilitate the transfer of patients to stroke centers, where advanced stroke care can be provided, ensuring that patients receive the best possible care.

Wearable technology has been used to monitor patients' vital signs and physical activity, enabling healthcare providers to quickly detect and respond to potential strokes. Remote monitoring allows healthcare providers to monitor patients' vital signs, such as blood pressure, heart rate, and oxygen saturation, from a distance. Mobile applications provide patients with access to rehabilitation exercises, medication reminders, and self-monitoring tools to track their progress. In a large review published in 2022 in The Lancet Digital Health, telemedicine consultations and remote monitoring using wearable technology improved medication adherence, blood pressure control, and health-related quality of life in cardiovascular patients. 12 Similar findings have been published in the literature. 13 In 2018, Apple launched a study to investigate whether the heart rate sensor on its Apple Watch (Apple Inc., Cupertino, CA, USA) could detect atrial fibrillation (AFib). The study involved over 400,000 participants and used an algorithm to analyze heart rate data for irregularities suggestive of AFib. 14 The results showed that the Apple Watch had a high degree of accuracy in detecting AFib, with a sensitivity of 71% and a specificity of 84%. 14 The study demonstrated the potential of wearable technology, such as the Apple Watch, to help screen for AFib and provide early detection of the condition, leading to earlier intervention and improved patient outcomes. 14 As a result of the study, Apple received U.S. Food and Drug Administration (FDA) clearance for its electrocardiogram (ECG) app, which allows users to take an ECG directly from their Apple Watch and detect AFib. In a comparison of many wearables, including some from famous brands such as Apple and Samsung (Samsung Group, Swon-si, South Korea), the authors stated a high accuracy of wearables in identifying suspected rhythms of AFib. 15

Automated neuroimaging interpretation has emerged as a promising tool for diagnosing and treating acute stroke. Neuroimaging, such as CT and magnetic resonance imaging (MRI), is critical to stroke diagnosis and management, providing detailed images of the brain and blood vessels. However, the interpretation of these images can be time-consuming and require specialized expertise. Automated neuroimaging interpretation tools use AI algorithms to analyze imaging data and identify potential stroke lesions, improving the accuracy and speed of diagnosis. On the other hand, this kind of technology is still expensive. Several studies have evaluated the effectiveness of automated neuroimaging interpretation in acute stroke, with promising results. 16 A study conducted by Straka et al. 16 showed that an automated software algorithm had a sensitivity of 92% and a specificity of 99% in detecting large vessel occlusion on CT angiography scans. The use of automated neuroimaging interpretation led to a significant reduction in time from symptom onset to treatment, resulting in improved outcomes for patients with acute stroke. A study 17 published in 2023 introduces an end-to-end learning approach for the automatic determination of collateral scores from CT angiography (CTA) images, which are crucial for treatment decision-making in acute stroke patients. The method involves preprocessing the CTA image to align it with an atlas and dividing it into affected and healthy hemispheres, followed by feature extraction using a VoxResNet-based convolutional neural network within a Siamese model framework. The findings suggest that end-to-end learning for collateral scoring is feasible, performs comparably to traditional methods, and can be integrated into existing functional outcome prediction models, indicating its potential utility in clinical settings. 17

Automated neuroimaging interpretation can potentially improve stroke diagnosis and treatment, enabling earlier intervention and better patient outcomes. 18 As automated algorithms continue to develop and then may become more accurate, their use in acute stroke care is likely to become more widespread, allowing for even faster diagnoses and treatments, and ultimately improving the lives of stroke patients. 18 19 Implementing these resources has many challenges, including the need for large datasets and the potential for bias in algorithm development. 19 Collaborations between radiologists, stroke specialists, and computer scientists can help overcome these challenges and accelerate the development and implementation of AI in acute stroke care. 19

Mobile applications and virtual reality-based telerehabilitation programs have been used to deliver personalized rehabilitation programs to stroke patients, improving their recovery outcomes. A randomized controlled trial published 20 in *JAMA Neurology* , in 2019, found that a virtual reality-based telerehabilitation program significantly improved upper limb motor function in stroke patients. 20 A study by Maier et al. 21 explored the effectiveness of a mobile app-based telerehabilitation program for poststroke patients. The results showed significant improvements in motor function, balance, and quality of life compared with traditional in-person therapy. 21

Virtual reality technology has gained importance in telerehabilitation as it provides an immersive and interactive experience for patients. 22 A virtual reality-based telerehabilitation utilizes headsets or motion-sensing devices to create virtual environments that simulate real-world scenarios, engaging patients in therapeutic exercises. 22 Research conducted by Perez-Marcos et al. 22 demonstrated the potential of this rehabilitation modality in stroke patients. The study reported significant improvements in upper limb motor function and cognitive performance among stroke patients who underwent virtual reality-based telerehabilitation. By simulating functional tasks and challenging environments, virtual reality promotes motor learning and neuroplasticity, enhancing rehabilitation outcomes. 22

Electronic health records (EHR) and data analytics have been used to collect and analyze stroke patient data, enabling healthcare providers to identify patterns and trends and improve patient outcomes. 23 The study by Yang et al. 24 developed an automated method to extract NIHSS (National Institutes of Health Stroke Scale) scores from EHR to aid in stroke-related clinical investigations. Utilizing a two-step pipeline approach and the MIMIC-III database, the method showed high accuracy, outperforming traditional rule-based methods. This approach facilitated the retrieval of structured scale data for clinical research in real-world settings. 24 Another study by Fonarow et al. (2010) 25 found that EHR adoption was associated with lower in-hospital mortality rates for stroke patients. The authors 25 describe a large-scale analysis from the Get With The Guidelines (GWTG)-Stroke program, which covered 1,000,000 patient admissions for various types of stroke and transient ischemic attack (TIA) from 1,392 U.S. hospitals between 2003 and 2009. The study 25 found notable variations in in-hospital mortality rates across different types of cerebrovascular events, with the highest mortality associated with intracerebral hemorrhage (25.0%) and subarachnoid hemorrhage (20.4%), followed by ischemic lowest stroke (5.5%), and the in TIA patients (0.3%). The study 25 also noted improvements in the length of hospital stays and a reduction in risk-adjusted in-hospital mortality for ischemic stroke and TIA patients. The clinical impact of these findings is significant, demonstrating that systematic quality improvement programs like GWTG-Stroke can lead to substantial enhancements in the care and outcomes of stroke and TIA patients.

Usually incorporated into EHR resources, the data analytics is a powerful tool in stroke care that allows for the analysis of large datasets to identify patterns and trends that can improve stroke care outcomes. Data analytics can be used to predict stroke risk, facilitate early diagnosis, and monitor treatment response. For instance, a study by Amarasingham et al. 26 showed that the use of data analytics helped reduce emergency department boarding times for stroke patients by 19%. Data analytics also provide insights into stroke care quality metrics, enabling healthcare providers to identify areas for improvement and implement evidence-based interventions to enhance care delivery. 26 27

**Figure 2:**
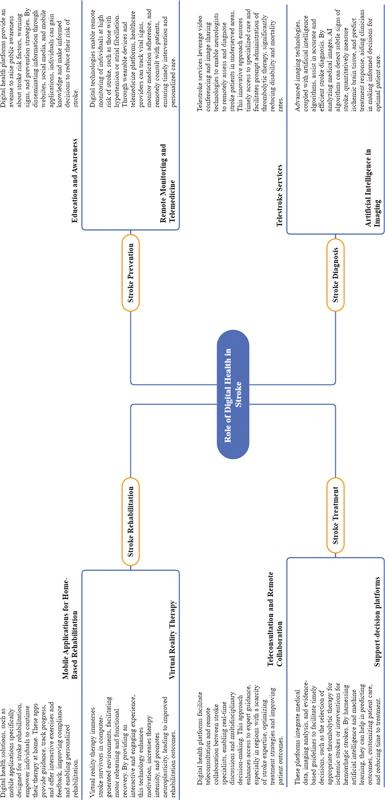
Note: Image generated with ChatMind. Mental map summarizing the role of digital health in vascular neurology.

In Figure 2 , we summarize digital health applications and their adoption in stroke field.

## CURRENT CHALLENGES PREVENTING DIGITAL HEALTH INTERVENTION IN STROKE

While there is great potential for digital health interventions in stroke medicine, several challenges must be addressed. One of the biggest challenges is limited access to technology, particularly among underserved and rural populations. 28 Digital health interventions require access to devices such as smartphones, computers, wearables, and internet connectivity. However, many stroke patients, particularly those from low-income and rural communities, may not have access to these resources, limiting the reach of digital health interventions. 28 29 These facts are related to the concept of “digital exclusion.” Digital exclusion in health refers to the disparity experienced by certain individuals or groups in accessing and benefiting from digital health technologies and services. 28 29 This phenomenon is often driven by factors such as socioeconomic status, age, literacy, geographic location, and disability, leading to a significant portion of the population being inadvertently marginalized in the rapidly evolving digital healthcare landscape. The implications of digital exclusion are profound, as it not only widens the health equity gap but also undermines the potential of digital health innovations to deliver universal and personalized care. Without concerted efforts to address digital exclusion, advancements in healthcare technology risk reinforcing existing disparities, thereby compromising the overarching goal of improving public health outcomes and ensuring that the benefits of digital health are accessible to all, irrespective of their background or circumstances. 29

Another challenge is ensuring the privacy and security of patient data. Digital health interventions rely on collecting and sharing patient data, including personal health information. However, ensuring the privacy and security of this data are critical to maintaining patient trust and preventing data breaches. Robust security measures, such as encryption and two-factor authentication, must be in place to protect patient data and comply with data protection regulations. In Brazil, for example, many EHR platforms were obliged to incorporate security features for dealing with, storing, and sharing sensitive data after the approval of a national law on the protection of personal data. 30 Moreover, challenges about the adoption of AI include issues around data privacy and security, ethical concerns around the use of this resource in medical decision-making, and the need for regulatory frameworks to ensure the safe and effective use of AI in healthcare. As AI technology continues to advance, it will be important to address these challenges to ensure that AI can be effectively integrated into stroke care and improve patient outcomes. 31

A third challenge is integrating digital health interventions with clinical workflows. Digital health interventions must be seamlessly integrated into existing clinical workflows to be effective. 32 However, this can be challenging due to the complexity of healthcare systems and the need for interoperability between different technologies. There is a need for greater collaboration between healthcare providers, technology companies, and policymakers to establish standards for interoperability and promote the seamless integration of digital health interventions into clinical practice. 32

Addressing these challenges will be critical to achieve the full potential of digital health interventions in stroke medicine. Efforts to increase access to technology, ensure data privacy and security, and promote integration with clinical workflows will be essential to expanding the reach and impact of digital health interventions and improving outcomes for stroke patients.

## FUTURE DIRECTIONS WITH AN EMPHASIS ON HOW DIGITAL HEALTH INTERVENTION CAN HELP SOLVE SPECIFIC PROBLEMS IN STROKE CARE

Digital health interventions can potentially address several specific problems in stroke care, leading to improved patient outcomes. Early detection and intervention are critical in stroke care, as timely treatment can reduce disability and improve outcomes. Digital health interventions, such as wearable devices and mobile applications, can help detect and monitor stroke risk factors, such as high blood pressure and irregular heartbeat. These interventions can also provide real-time feedback to patients and healthcare providers, enabling earlier intervention and preventing stroke.

We strongly believe the AI has the potential to revolutionize stroke care by improving efficiency, accuracy, and outcomes. In the future, as we said, AI could be used to develop predictive models that identify patients at high risk of stroke and personalize their treatment plans. Artificial intelligence could also be used to interpret complex neuroimaging data and provide real-time diagnostic support to stroke care teams. 33

Stroke can cause long-term disability and impairments, requiring extensive rehabilitation and support. Digital health interventions, such as virtual reality and telerehabilitation, can provide innovative stroke rehabilitation and recovery solutions. Virtual reality can simulate real-life scenarios to help patients practice and regain motor skills. At the same time, telerehabilitation can provide remote access to rehabilitation services, increasing access to care for underserved populations. 33

Patient education and empowerment are critical in stroke prevention and management. Digital health interventions can provide patients with easy access to educational materials, such as videos and interactive tools, to help them understand their condition and take an active role in their care. These interventions can also provide real-time feedback to patients, enabling them to track their progress and make informed decisions about their health. 34

Regarding the adoption of complex algorithms for assessing diagnoses, making predictions or prognostications, 24 25 or performing neuroimaging diagnoses, 16 17 18 19 the realm of AI applications demands meticulous selection and preprocessing of variables to ensure the robustness and reliability of the resulting algorithms. 24 This process typically involves a series of methodologically rigorous steps designed to enhance the predictive accuracy and generalizability of AI models. 27 31 The initial phase in the development of a healthcare AI application involves the careful selection of relevant variables. This selection is driven by the specific clinical question or healthcare problem being addressed. Variables are chosen based on their proven or hypothesized relevance to the outcomes of interest, informed by existing clinical knowledge and prior research findings. 31 This step is crucial, as the inclusion of irrelevant or redundant variables can lead to model overfitting and decreased generalizability. After the AI model is developed, it is imperative to validate its performance using an independent cohort that was not involved in the model training phase. This validation is critical for assessing the model's ability to generalize to new, unseen data, which is a hallmark of a robust AI application. Validation involves comparing the model's predictions against actual outcomes using performance metrics such as accuracy, sensitivity, specificity, and area under the receiver operating characteristic curve (AUC-ROC). The independent cohort should ideally be diverse and representative of the population where the AI application is intended to be used. This approach not only affirms the efficacy of the model across different subgroups but also identifies potential limitations or biases in the model. 31

Future directions in digital health interventions for stroke care are poised to address some of the most pressing challenges in the field, including the need for timely diagnosis, personalized rehabilitation programs, and ongoing patient support outside the clinical setting. 33 34 35 Advancements in AI and data analytics hold the potential to revolutionize stroke diagnosis by enabling the rapid interpretation of imaging studies, which is crucial for initiating time-sensitive treatments such as thrombolysis. Moreover, digital platforms can facilitate tailored rehabilitation programs by leveraging virtual reality and gamification, thereby enhancing patient engagement and adherence to treatment plans. 36 These interventions can also support continuous monitoring and adjustment of treatment regimens based on real-time patient data, potentially leading to improved outcomes in terms of recovery speed and quality of life poststroke.

However, the integration of digital health technologies in stroke care is not without its challenges, particularly in the realms of regulation and ethics. 35 36 37 Ensuring the privacy and security of patient data are paramount, as these technologies often involve the collection and analysis of sensitive health information. Regulatory frameworks must evolve to keep pace with technological advancements, ensuring that digital health interventions are not only effective but also safe and compliant with data protection laws. 38 39 40 41 Additionally, ethical considerations related to patient autonomy, informed consent, and equity in access to these technologies must be addressed to prevent disparities in care. Addressing these regulatory and ethical challenges is essential for building trust in digital health solutions and ensuring their sustainable integration into stroke care practices. 42 43 44 45

In conclusion, digital health is set to transform stroke care by enhancing prevention, diagnosis, treatment, and rehabilitation. Telestroke programs are reducing treatment times, boosting clinical outcomes, and broadening access to specialized care. Meanwhile, wearable tech and apps offer tailored rehab programs and remote monitoring, enhancing recovery and adherence to treatment. Innovations in automated neuroimaging tools are also speeding up and refining stroke diagnoses, leading to earlier interventions. Ensuring these technologies are validated, safe, and universally accessible is crucial for benefiting all stroke patients, irrespective of their background or location. The future of digital health interventions in stroke care is incredibly promising, with innovations aimed at enhancing diagnosis, personalized rehabilitation, and patient support. Technologies like machine learning are set to transform the speed and accuracy of stroke diagnosis, crucial for timely treatments. Digital platforms, through virtual reality and gamification, are expected to make rehabilitation programs more engaging, improving adherence and potentially speeding up recovery. However, the successful integration of these technologies must navigate regulatory and ethical hurdles, ensuring data privacy, security, and equitable access to prevent care disparities.
